# ﻿A new species of the *Spirobranchuskraussii* complex, *S.akitsushima* (Annelida, Polychaeta, Serpulidae), from the rocky intertidal zone of Japan

**DOI:** 10.3897/zookeys.1100.79569

**Published:** 2022-05-12

**Authors:** Eijiroh Nishi, Hirokazu Abe, Katsuhiko Tanaka, Naoto Jimi, Elena K. Kupriyanova

**Affiliations:** 1 College of Education, Yokohama National University, Hodogaya, Yokohama 240-8501, Japan Yokohama National University Yokohama Japan; 2 Department of Biology, Center for Liberal Arts & Sciences, Iwate Medical University, Idaidori 1-1-1, Yahaba, Shiwa-gun, Iwate 028-3694, Japan Iwate Medical University Yahaba Japan; 3 Current address: Department of Biological Sciences, Faculty of Science and Engineering, Ishinomaki Senshu University, Shinmito 1, Minamisakai, Ishinomaki, Miyagi 986-8580, Japan Ishinomaki Senshu University Ishinomaki Japan; 4 Department of Marine Biology, School of Marine Science and Technology, Tokai University, 3-20-1, Orido, Shimizu, Shizuoka-shi, Shizuoka 424-8610, Japan Tokai University Shimizu Japan; 5 Sugashima Marine Biological Laboratory, Graduate School of Science, Nagoya University, 429-63 Sugashima, Toba, Mie 517-0004, Japan Nagoya University Toba Japan; 6 Centre for Marine and Coastal Studies, Universiti Sains Malaysia 11800 USM, Penang, Malaysia Universiti Sains Malaysia Penang Malaysia; 7 Australian Museum Research Institute, Australian Museum, 1 William Street, Sydney 2010 NSW, Australia Australian Museum Sydney Australia; 8 Department of Biological Sciences, Macquarie University, North Ryde NSW 2109, Australia Macquarie University North Ryde Australia

**Keywords:** Cosmopolitan species, paleo-aggregation, sea level indicator

## Abstract

A new species of *Spirobranchus* (Annelida: Serpulidae) is described based on specimens collected at the coastal Shonan area of Sagami Bay and the adjacent areas of Honshu, Japan. *Spirobranchusakitsushima***sp. nov.** forms large aggregations in the intertidal rocky zone of warm-temperate Japanese shores. This species was referred to as *Pomatoleioskraussii* (Baird, 1864) until the monotypic genus *Pomatoleios* was synonymized with *Spirobranchus*. This new species is formally described based on morphologically distinct Japanese specimens with supporting DNA sequence data. The calcareous opercular endplate of *Spirobranchusakitsushima***sp. nov.** lacks a distinct talon, but some specimens have a slight rounded swelling on the endplate underside, while in other species of the *S.kraussii* complex a talon is present, usually extended, and with bulges. We examined sub-fossil tube aggregations of the new species and suggest that such aggregation stranded ashore is a good indicator of vertical land movements (uplift and subsidence) resulting from past events, such as earthquakes, in Honshu, Japan.

## ﻿Introduction

The family Serpulidae Rafinesque, 1815 is a unique and distinct group of marine annelids that inhabits self-secreted calcareous tubes and is recorded in all habitats of the world oceans, from the intertidal zone, shallow-water coral reefs to abyssal and hadal depths, as well as in brackish and freshwater habitats. Currently, the family comprises 562 valid species in 69 genera (Capa et al. 2021). The most speciose genus of the family is *Hydroides* Gunnerus, 1768, with more than 100 species; *Spirobranchus* de Blainville, 1818 is the second largest genus with 36 nominal species (Capa et al. 2021; Tables 3, 4).

Approximately 70 serpulid species have been recorded in Japanese waters (Nishi et al. 2017). Among them, 11 are species of the genus *Spirobranchus*, while the morphospecies *S.cruciger* (Grube, 1862) and *S.gaymardi* Quatrefages, 1866 are considered synonyms of *S.corniculatus* (Grube, 1862) based on a recent genetic study (Willette et al. 2015). The group of species commonly known as Christmas Tree Worms is the most conspicuous in the genus *Spirobranchus* because of its brilliantly colored spiral radiolar crowns. These large-bodied species (e.g., *S.corniculatus* and *S.gardineri* Pixell, 1913 in the Pacific) are associated with hermatypic corals in warm temperate to tropical waters of Japan.

Another well-known species of *Spirobranchus* is distributed in temperate to sub-tropical Japanese coastal areas from Honshu to Kyushu, and in the vicinity of the Nansei Archipelago. This species is known in Japan under the common name “Yakko-kanzashi Gokai” because the ventral side of its opercular peduncle has two dark lateral bands on a white background, which makes it look like “Yakko”: this Japanese word describes a unique hairstyle (or a person with such a hairstyle) with a shaved top of the head and hair around the ears cut in the shape of a plectrum (pick) used for Samisen, a traditional Japanese stringed instrument (Otsuki 1935). As “Kanzashi” is a Japanese word for an ornamental hairpin and “Gokai” means a polychaete worm, then “Kanzashi-Gokai” is a Japanese common name for serpulid polychaetes.

“Yakko-kanzashi” is a gregarious species commonly forming distinct intertidal belts along with barnacles and bivalves. Morphologically, the specimens of “Yakko-kanzashi” are characterized by opercula covered with simple endplates, arrangement of radioles in two semi-circles, absence of collar chaetae in adults, and tough thick-walled blue or purple tubes with sharp or flattened keels. This species has been recorded under a number of scientific names. Initially it was referred to (e.g., Okuda 1937, 1940; Utinomi 1956) as *Pomatoleioscrosslandi* Pixell, 1913, a species originally described from off Tanzania. After *P.crosslandi* was synonymized with *Pomatoleioskraussii* (Baird, 1864), the attribution of the Japanese population changed accordingly (e.g., Imajima and Hartman 1964; Okuda and Imajima 1965; Uchida 1992). Most recently it was referred to as *Spirobranchuskraussii* (e.g., Nishi et al. 2017) because the genus *Pomatoleios* was synonymized with *Spirobranchus* by Pillai (2009). The reported range of the nominal species spans in Japan from northern Honshu to tropical Okinawa (e.g., Onagawa Bay: Okuda 1937; Okinawa: Okuda 1940; Nishi 1993; Sagami Bay: Imajima 1968; Wakayama, Izu, Kochi: Uchida 1978). Some studies examined its distribution (Nishi 1993), early development (Sawada 1988), and life history (Miura and Kajihara 1984).

The assignment of the Japanese *Spirobranchus* “Yakko-kanzashi” to the morphologically similar intertidal belt-forming *Spirobranchuskraussii* was based on the wide distribution attributed to *S.kraussii*. After its original description from warm-temperate coasts of South Africa, the taxon was subsequently reported from numerous tropical and subtropical localities (Persian (Arabian) Gulf, Pakistan, Sri Lanka, Philippines, Hawaii, Australia, China (including Hong Kong), Japan, Korea, Singapore, Suez Canal, and eastern Mediterranean, see Simon et al. 2019). However, such wide, nearly cosmopolitan distributions were recently questioned (Hutchings and Kupriyanova 2018). Genetic studies revealed that this warm temperate intertidal species is restricted to South African coasts and that taxa under this name from other areas belong to a large complex of regionally distributed species (Simon et al. 2019; Pazoki et al. 2020; Sivananthan et al. 2021).

Two specimens collected in Japan from Manazuru, Sagami Bay, Honshu and deposited in the Australian Museum (AM W.49980 and AM W.49981) were sequenced and used in the study of Simon et al. (2019). The sequences formed a distinct genetic lineage denoted as *Spirobranchus* sp. 1 by Simon et al. (2019) providing evidence supporting the presence of an undescribed species of the *S.kraussii* complex in Japan. The most recent genetic study by Kobayashi and Goto (2021) recovered three genetic lineages within the *S.kraussii* complex in Japan, which suggests that there are at least three unnamed species in Japan: *S.* sp. 1 from warm temperate localities (Seto, Wakayama), and two from tropical Okinawa (*Spirobranchus* spp. 5 and 6).

Serpulids forming intertidal belts and relics of such assemblages are useful fixed biological indicators (FBIs) as they provide data on short-term fluctuations in sea-level (Baker et al. 2001b). A belt-forming Australian serpulid *Galeolariacaespitosa* Lamarck, 1818 was used as a marker species in relative sea-level height analyses of past environmental changes (Bird 1988; Baker et al 2001a, b). The height differential of fossil to living encrustations is a simple and reliable measure of changes on tectonically stable coasts of eastern Australia (Baker and Haworth 1997). Japanese “Yakko-kanzashi”, occupying intertidal habitats similar to those of *G.caespitosa*, is a useful paleoindicator of sea-level changes caused by tectonic events, such as earthquakes. While current aggregations are always found at the sea level, paleo-aggregations are stranded ashore far above it. In Tanabe Bay, Kii Peninsula, current aggregations had the upper limit of +0.1 – +0.2 m from the mean sea level (MSL) (Nishimura 1972). Kayanne et al. (1987) defined dense aggregation of tubes as “almost 100% of areas of 10 cm^−2^ were covered by serpulid tubes”, and they reported a similar upper limit (+0.1 to ± 0.1 m from MSL) of dense aggregations found on Boso Peninsula, Chiba. Comparisons of Nishibata et al. (1988) revealed the upper limit of the current population (= dense aggregation) as +12–± 2 cm from MSL, while that of the fossil ones ranged from +68 to +235 cm. Nishibata et al. (1988) showed that paleo-aggregations at the site of Taisho-Kanto great earthquake in A.D. 1923 were located 1.2–1.4 m above MSL, while those found in the vicinity of Genroku-Kanto great earthquake in A.D. 1703 raised to 2.3 m above MSL. Similarly, Maemoku and Tsubono (1990), Shishikura (2003a) and Shishikura et al. (2007) used uplifted paleo-aggregations to reconstruct the earthquake history along Miura, Boso and Kii Peninsula, Honshu. In Muroto, Kochi, Maemoku (2001) estimated that the older tube aggregations uplifted to 8.3–9.1 m between 2800 and 4500 years ago as a result of an earthquake.

The main aim of this study is to formally describe and name the common intertidal gregarious species of Sagami Bay and adjacent areas previously referred to as *S.kraussii*, using a combination of morphological and molecular data. We also examine and describe in detail paleo-aggregations (stranded ashore and rarely overlapping with the current tube aggregations) of this species.

## ﻿Materials and methods

**Figure 1.**
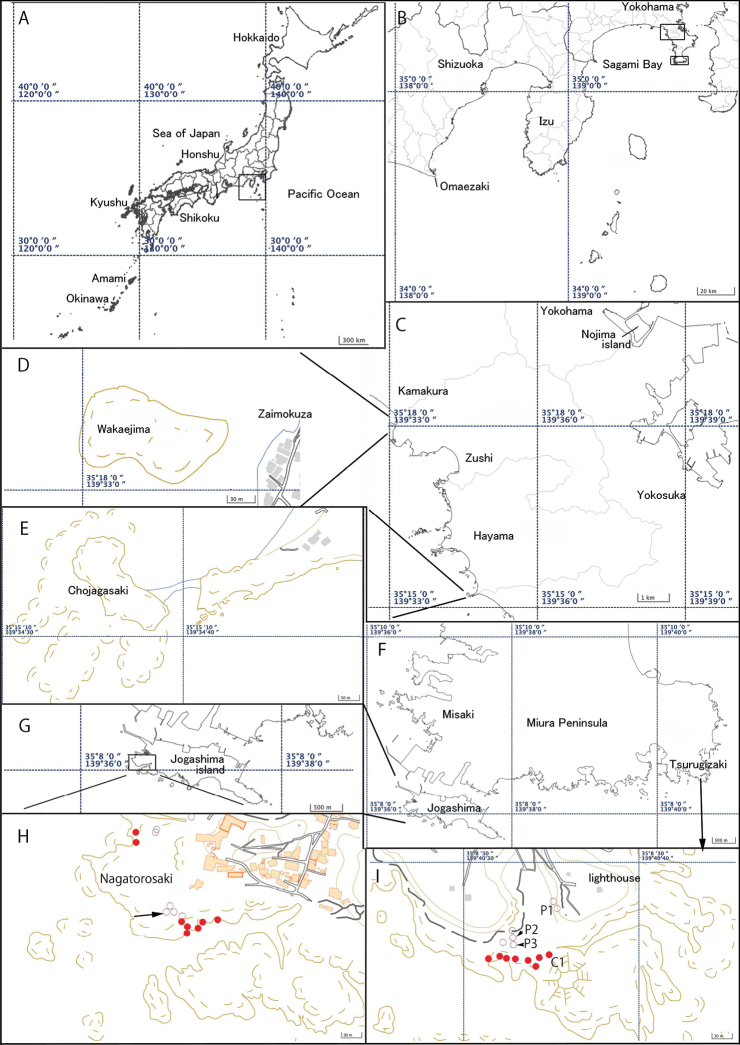
Map of collection sites **A** Japan and adjacent seas **B** Sagami Bay, Suruga Bay, and Pacific side of Honshu, and collection sites on Miura Peninsula and Yokohama **C** collection sites of Miura Peninsula and Yokohama **D** Wakaejima, Kamakura, type locality of *Spirobranchusakitsushima* sp. nov. **E** Hayama **F** Western part of Miura Peninsula, showing Tsurugizaki and Jogashima **G** Jogashima **H** close-up view of collection sites of Jogashima **I** close-up view of Tsurugizaki. Key: ○: paleo-aggregation, ●(red): current distribution. Arrow in **H** indicates (**A**) in Fig. 3; P1–P3 and C1 in **I** indicates site of (**I**) and (**J**) in Fig. 3.

Specimens were collected around Sagami and Suruga Bay (Fig. 1A, B) and specimens from Chichijima Island, Ogasawara were added for a comparison. The specimens designated as types were collected in Wakaejima, Kamakura, Sagami Bay (Fig. 1C, D). Current and paleo- tube aggregations of the species were photographed (and some tubes were collected) at Tsurugizaki and Jogashima (Fig. 1F–I) and altitudes of their aggregations were compared to current MSL.

The holotype, paratypes and additional specimens were deposited in the Natural History Museum and Institute, Chiba (**CBM-ZW**), Japan, the Coastal Branch of Natural History Museum and Institute, Chiba (**CMNH-ZW**), Katsuura, Chiba, and Marine Science Museum, Tokai University (**MSM-INV**), Shimizu, Shizuoka, Japan. Two specimens are deposited in the Australian Museum (**AM**) (AM W.49980 and AM W.49981).

Terminology for voucher specimens used to produce molecular samples was used following Pleijel et al. (2008). Hologenophore is a specimen voucher from which the molecular sample is derived, paragenophore is a putatively conspecific voucher specimen collected together with the ‘molecular specimen’, and syngenophore is a voucher collected at another place or time.

A total of 14 worms for which DNA has been sequenced (hologenophores sensu Pleijel et al. 2008) were preserved in 75% ethanol. Some paratypes and non-type specimens were anesthetized with magnesium hydroxide and photographed alive or after being fixed in 10% formalin seawater. In order to examine the morphology of the lower endplate surface (presence of the talon and its shape), endplates were taken out from the opercular tissue using scalpel and forceps.

For scanning electron microscopy (SEM) observation specimens were dehydrated through gradual series of ethanol for 10 min in each and finally washed with 100% ethanol for 10 min. The samples then were washed with 1:1 and 1.5:0.5 mixture of 100% ethanol and hexamethyldisalazane (HMDS) for 10 minutes in each, and finally washed with 100% HMDS for 10 min following Nation (1983) and Wang et al. (2018). Specimens were left overnight to ensure HMDS evaporation, then coated with platinum and viewed under a JEOL JF7001FM at the Instrumental Analysis Center of Yokohama National University.

The partial sequences of the mitochondrial cytochrome b (cytb) gene, nuclear internal transcribed spacer-2 (ITS2) region, and 18S and 28S rRNA genes were used for comparisons with congeneric species. Genomic DNA was extracted from posterior abdomens of ethanol-fixed worms collected from the Shonan area (Sagami Bay) and from Omaezaki (Suruga Bay) (Table 1) by heating at 96 °C for 20 min in 50 μl of TE buffer with 10% Chelex 100 (Bio-Rad Laboratories, Hercules, CA) according to Richlen and Barber (2005). Undiluted or 10-fold diluted DNA extract was used as a template for polymerase chain reaction (PCR). The 10 μL reaction mix contained 7.05 μL of sterilized water, 0.05 μL of TaKaRa Ex Taq Hot Start Version (TaKaRa Bio, Kusatsu, Japan), 1.0 μL of 10× Ex Taq Buffer, 0.8 μL of 2.5 μM dNTP mixture, 0.05 μL of 50 μM of each forward and reverse primers, and 1.0 μL of template DNA for the mitochondrial cytb gene and the nuclear ITS2 region. The 25 μL reaction mix contained 11.3 μL of sterilized water, 12.5 μL of 2 × KOD One PCR Master Mix (TOYOBO, Osaka, Japan), 0.1 μL of 50 μM each of forward and reverse primers, and 1.0 μL of template DNA for nuclear 18S rRNA gene. The 10 μL reaction mix contained 4 μL of sterilized water, 5 μL of 2 × KOD One PCR Master Mix (TOYOBO, Osaka, Japan), 0.05 μL of each 50 μM forward and reverse primers, and 1.0 μL of template DNA for nuclear 28S rRNA gene.

**Table 1.** Collection information, GenBank accession numbers of specimens used in this study and references. The type specimens of the new Japanese species are deposited in the Natural History Museum and Institute, Chiba, Japan (CBM).

Species	Locality	Accession number				Museum voucher	Reference
		cytb	ITS2	18S	28S		
*S.akitsushima* sp. nov.	Kamakura, Japan	LC661622	LC661636	LC661650	LC661664	CBM-ZW 1127 (holotype)	This study
	Kamakura, Japan	LC661623	LC661637	LC661651	LC661665	CBM-ZW 1128	This study
	Kamakura, Japan	LC661624	LC661638	LC661652	LC661666	CBM-ZW 1129	This study
	Kamakura, Japan	LC661625	LC661639	LC661653	LC661667	CBM-ZW 1130	This study
	Kamakura, Japan	LC661626	LC661640	LC661654	LC661668	CBM-ZW 1131	This study
	Omaezaki, Japan	LC661627	LC661641	LC661655	LC661669	CBM-ZW 1132	This study
	Omaezaki, Japan	LC661628	LC661642	LC661656	LC661670	CBM-ZW 1133	This study
	Omaezaki, Japan	LC661629	LC661643	LC661657	LC661671	CBM-ZW 1134	This study
	Kamakura, Japan	LC661630	LC661644	LC661658	-	CBM-ZW 1135	This study
	Kamakura, Japan	LC661631	LC661645	LC661659	-	CBM-ZW 1136	This study
	Kamakura, Japan	LC661632	LC661646	LC661660	-	CBM-ZW 1137	This study
	Kamakura, Japan	LC661633	LC661647	LC661661	-	CBM-ZW 1138	This study
	Kamakura, Japan	LC661634	LC661648	LC661662	-	CBM-ZW 1139	This study
	Kamakura, Japan	LC661635	LC661649	LC661663	-	CBM-ZW 1140	This study
	Manazuru, Japan	MK308653	-	MK308668	-	AM W.49980	Simon et al. (2019)
	Manazuru, Japan	MK308654	-	MK308669	-	AM W.49981	Simon et al. (2019)
	Shirahama, Japan	LC604687	LC604683	-	-	-	Kobayashi and Goto (2021)
	Shirahama, Japan	LC604688	LC604684	-	-	-	Kobayashi and Goto (2021)
*S.aloni*	Israel	MF319301	MF319230	MF319276	-	VR.25186	Perry et al. (2018)
*S.bakau*	Singapore	MW767145	-	MW767153	-	ZRC.ANN.0480	Sivananthan et al. (2021)
*S.cariniferus*	New Zealand	JX144878	-	JX144817	-	-	Smith et al. (2012)
	New Zealand	MK775646	-	MK775626	MK775605	-	Gosselin et al. (2019)
*S.corniculatus*	Israel	MF319311	MF319244	MF319281	-	VR.25242	Perry et al. (2018)
	Philippines	KP892811	KP892792	KP892778	-	-	Willette et al. (2015)
	Qld, Australia	KP892795	KP892782	KP892774	-	-	Willette et al. (2015)
	Qld Australia	-	-	EU19538	EU195366	SAM E3608	Kupriyanova et al. (2009)
*S.gardineri*	Israel	MF319337	MF319262	MF319297	-	VR.25314	Perry et al. (2018)
*S.giganteus*	Brazil	NC032055	-	-	-	-	Seixas et al. (2017)
*S.kraussii*	South Africa	MK308650	-	MK308665	-	AM W.49991	Simon et al. (2019)
*S.lamarcki*	France	-	-	DQ140404	EU195354	ZMA V.Pol.5241	Lehrke et al. (2007)
*S.latiscapus*	New Zealand	JX144879	-	JX144821	-	-	Smith et al. (2012)
*S.lima*	France	-		DQ317130	EU256547	SAM E3538	Kupriyanova et al. (2006, 2009)
*S.sinuspersicus*	Iran	MN372436	-	MN372443	-	ZUTC.6808	Pazoki et al. (2020)
*S.taeniatus*	SA, Australia	-	-	DQ317120	EU195353	SAM E3532	Kupriyanova et al. (2006, 2009)
*S.tetraceros*	NSW, Australia	MN631161		-	-	AM W.42389	Palero et al. (2020)
S.cf.tetraceros	Israel (Red Sea)	MF319335	MF319257	MF319295	-	VR.25311	Perry et al. (2018)
	Spain (Mediterranean)	MN631163	-	-	-	MUVHN-ZK0002	Palero et al. (2020)
*S.triqueter*	Sweden	-		DQ317121	EU195348	SAM E3534	Kupriyanova et al. (2006, 2009)
*S.* sp. 2	Hawaii, USA	MK308655	-	MK308670	-	AM W.45327	Simon et al. (2019)
*S.* sp. 3	Qld, Australia	MK308647	-	MK308662	-	AM W.48301	Simon et al. (2019)
*S.* sp. 5	Yagachi Island, Japan	LC604689	LC604681	LC604685	-	-	Kobayashi and Goto (2021)
*S.* sp. 6	Oura Bay, Japan	LC604691	LC604679	LC604686	-	-	Kobayashi and Goto (2021)
*Galeolariahystrix*	New Zealand	JX144861	-	JX144799	-	-	Smith et al. (2012)
	SA, Australia	EU200441	-	DQ314839	EU256550	SAM E3526	Kupriyanova et al. (2006, 2009)
*Galeolariagemineoa*	NSW, Australia	FJ646535	FJ646551	-	-	SAM E3721	Halt et al. (2009)

The primer pairs used for PCR amplifications and sequencing are listed in Table 2. The PCR cycling conditions were (1) initial denaturation at 94 °C for 120 s followed by 35–45 cycles of denaturation at 94 °C for 30 s, annealing at 45 (for cytb) or 50 °C (for ITS2) for 40 s, and extension at 72 °C for 20 s, and a final extension at 72 °C for 300 s (TaKaRa Ex Taq), (2) 36 cycles of 98 °C for 10 s, 58 °C for 5 s, and 68 °C for 2 s for 18S rRNA gene (KOD One PCR Master Mix), and (3) 32 or 36 cycles of 98 °C for 10 s, 62 °C for 5 s, and 68 °C for 1 s for 28S rRNA gene (KOD One PCR Master Mix). The PCR products were purified using EnzSAP PCRClean-up Reagent (EdgeBio, San Jose, CA) and sequenced by Eurofins Genomics (Tokyo, Japan). The forward and reverse complementary sequences and contigs were assembled using GeneStudio ver. 2.2.0.0 (GeneStudio, Inc., Suwanee, GA). The obtained sequences have been deposited in the DDBJ/ENA/GenBank databases with accession numbers LC661622–LC661671 (Table 1). Intra-specific pairwise genetic distances (p-distance) for cytb sequences of *Spirobranchus* species were determined using MEGA version 11 software under default settings (Tamura et al. 2021).

**Table 2.** Primer pairs used for PCR amplifications and sequencing.

Gene	Primer	Direction	Sequence (5’–3’)	Usage	Reference
Cytb	cytb-spiroF	Forward	TATTGRGGKGCTACYGTWATTAC	PCR/Sequencing	This study
	cobr825	Reverse	AARTAYCAYTCYGGYTTRATRTG	PCR/Sequencing	Burnette et al. (2005)
ITS	ITS3	Forward	GCATCGATGAAGAACGCAGC	PCR/Sequencing	White et al. (1990)
	ITS4	Reverse	TCCTCCGCTTATTGATATGC	PCR/Sequencing	White et al. (1990)
18S	18S-1F	Forward	AACCTGGTTKATCCTGCCAGTAGTC	PCR/Sequencing	This study
	18S-1R654	Reverse	CAACTACGAGCTTTTTAACTGCAAC	Sequencing	This study
	18S-2F594	Forward	GCGGTAATTCCAGCTCCAATAG	Sequencing	This study
	18S-2R1233	Reverse	GAGTTTCCCCGTGTTGAGTC	Sequencing	This study
	18S-3F1153	Forward	CTGAAACTTAAAGGAATTGACGGA	Sequencing	This study
	18S-R1772	Reverse	TCACCTACGGAAACCTTGTTACG	PCR/Sequencing	Nishitani et al. (2012)
28S	D1R	Forward	ACCCGCTGAATTTAAGCATA	PCR/Sequencing	Scholin et al. (1994)
	D2C	Reverse	CCTTGGTCCGTGTTTCAAGA	PCR/Sequencing	Scholin et al. (1994)

Phylogenetic analyses based on concatenated gene sequences (cytb + ITS2 + 18S + 28S) and sequences of each gene/region were conducted using the sequences obtained in the present study supplemented with those sourced from DDBJ/ENA/ GenBank databases (Table 1). The sequences of *Galeolariahystrix* Mörch, 1863 and *G.gemineoa* Halt, Kupriyanova, Cooper & Rouse, 2009 were used as outgroups. The sequences of each gene/region were aligned using the MAFFT online service ver. 7 with the L‐INS‐i algorithm (Katoh et al. 2019). Ambiguously aligned regions of alignments were eliminated by employing Gblocks server ver. 0.91b (Castresana 2000) with the following less stringent settings: minimum number of sequences for a conserved/flank position were half the number of sequences + 1, maximum number of contiguous non-conserved positions was eight, minimum length of a block was five, and with half of the allowed gap positions. The final lengths of the alignments were 359 (cytb), 528 (ITS2), 1717 (18S), and 774 (28S) bp for the multiple sequence alignment.

Maximum likelihood (ML) analyses performed using IQ-TREE (Nguyen et al. 2015) implemented in PhyloSuite under Edge-linked partition model. For the concatenated dataset, the HKY+F+I+G4, K2P+I, TNe+I and TIM3+F+G4 models were selected for the cytb, ITS2, 18S and 28S rRNA gene/regions, respectively as the best-fit substitution model by ModelFinder (Kalyaanamoorthy et al. 2017) as implemented in IQ-TREE under the Bayesian information criterion (BIC). For the single gene/region data, the K3Pu+F+I+G4, K2P+G4, TNe+I and TIM3+F+G4 models were selected for the cytb, ITS2, 18S and 28S rRNA gene/region respectively. The robustness of the ML trees was evaluated by the Shimodaira-Hasegawa-like approximate likelihood-ratio test (SH-aLRT) with 5,000 replicates (Guindon et al. 2010), approximate Bayes (aBayes) test (Anisimova et al. 2011), and ultrafast bootstraps (UFBoot) with 5000 replicates (Hoang et al. 2018).

## ﻿Results

### ﻿Taxonomy

#### ﻿ Spirobranchus

Taxon classificationAnimaliaSabellidaSerpulidae

de Blainville, 1818

B2CE61F8-D36A-57C1-84D3-351DC9E4C9BD

##### Type species.

*Serpulagigantea* Pallas, 1766.

#### ﻿ Spirobranchus akitsushima sp. nov.

Taxon classificationAnimaliaSabellidaSerpulidae

7D33580A-0476-5755-AEB2-EDF5D93B9C26

http://zoobank.org/C79A1ACE-8027-4EC4-9854-FD1539F88956

**Figure 2.**
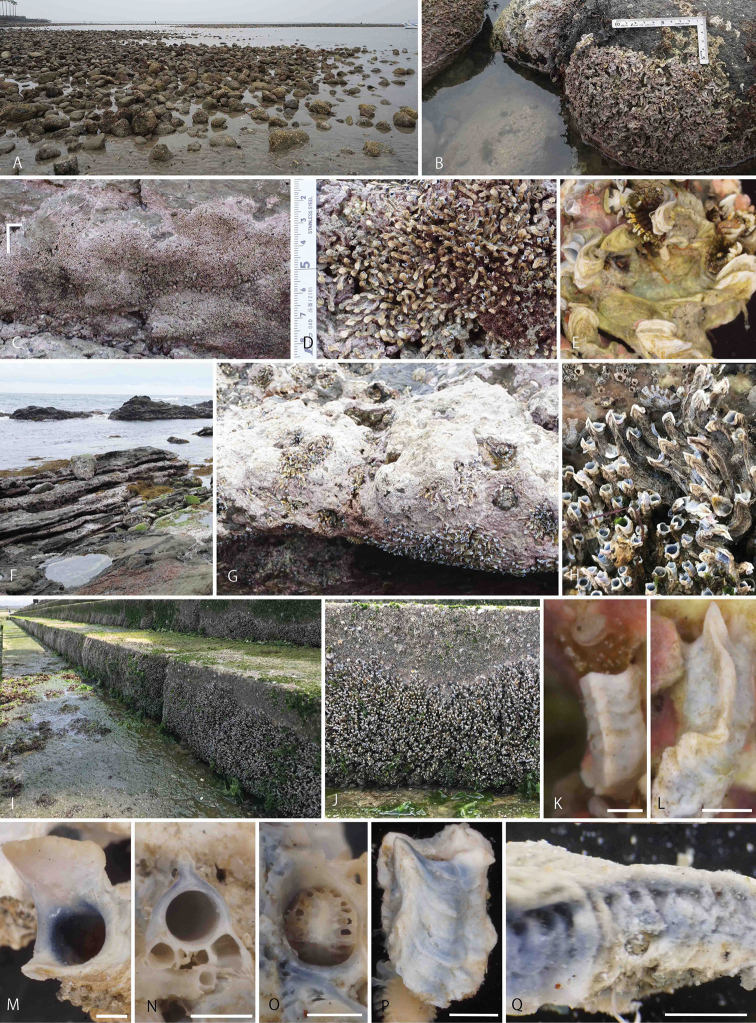
Field view of collection sites, aggregation, tubes of *Spirobranchusakitsushima* sp. nov. **A, B** Wakaejima, Kamakura **C–E** Hayama, Sagami Bay **F, G** Tsurugizaki, Miura Peninsula **H–J** Nojima, Yokohama. Aggregation of Yokohama found on concrete wall (**I**), around mean-sea level, thickness ~ 3–5 cm (**H, J**) **K–O** tubes of Kamakura population **P, Q** Ogasawara specimens. Scale bars: 1 mm (**K, L, M, N, O, P**), 2 mm (**Q**).

**Figure 3.**
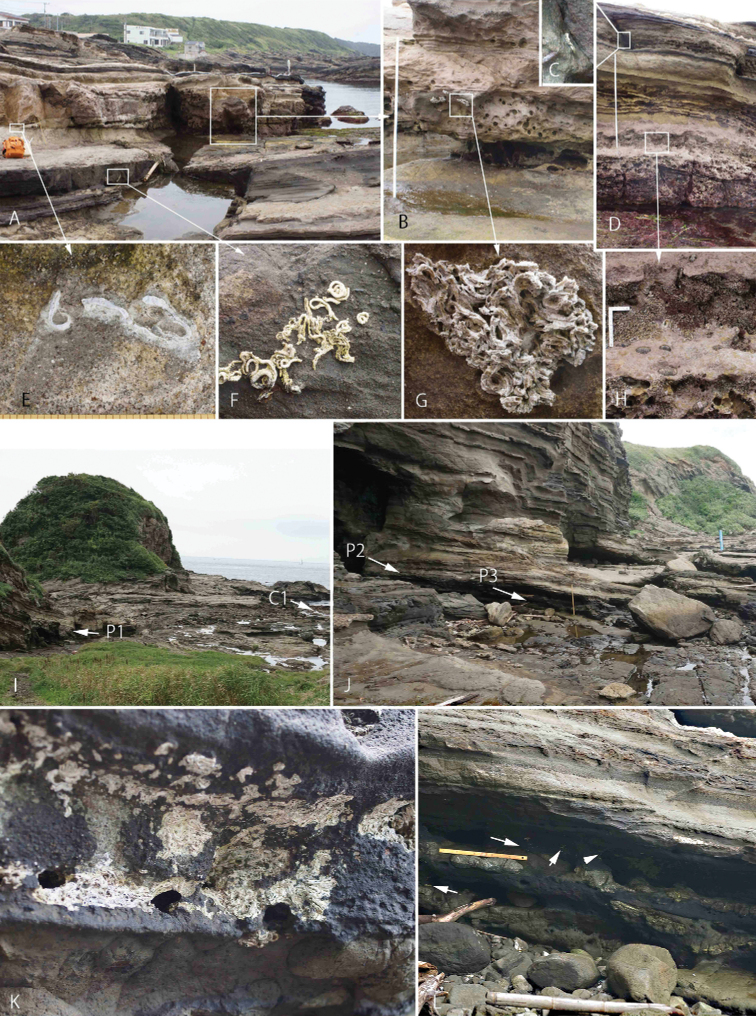
Paleo-aggregations of *Spirobranchusakitsushima* sp. nov. Jogashima (**A–H**), and Tsurugizaki (**I–L**) **K** close-up view of P2 of J **L** close-up of P3 of J. Paleo-aggregation (P1–3) and current distribution (C1) in **I** and **J** are corresponding to P1–P3 and C1 in Fig. 1I.

**Figure 4.**
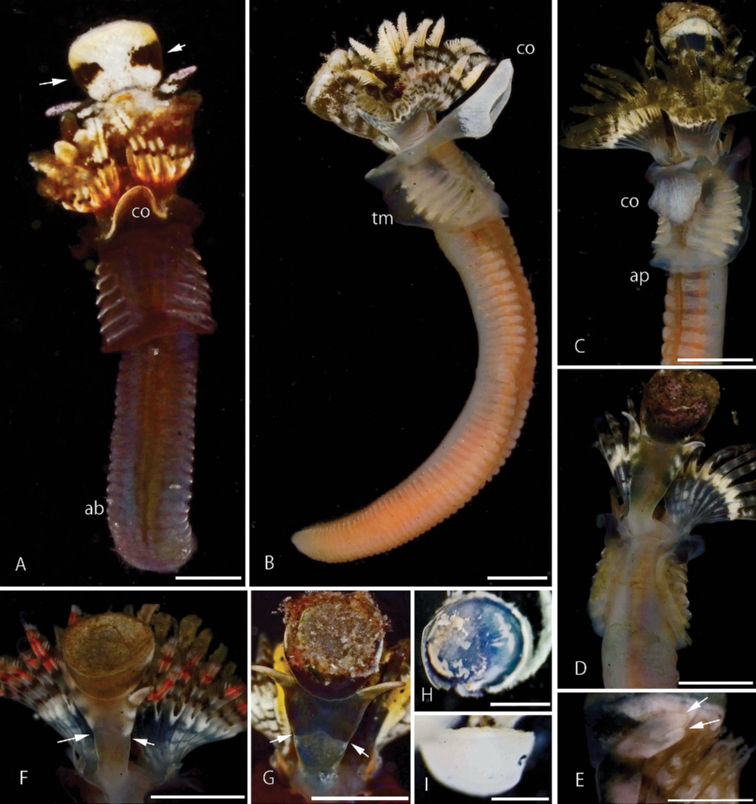
Type and non-type specimens of *Spirobranchusakitsushima* sp. nov. **A–E, H, I** Kamakura specimens **F** Hayama specimens **B, C** mature female specimen **A** ventral view, lateral band of peduncle (arrows) **D, F, G** dorsal view **B, E** lateral view **E** bilobed wing tip (arrows) **F, G** operculum in dorsal view, middle constriction (arrows) **H, I** dissected endplate **H** lower view, covered with a blue membrane **I** lateral view. Abbreviations: ac, achaetous chaetiger; ap, apron; c, collar; pd, peduncle; w, wing. Scale bars: 1 mm (**A, B**); 2 mm (**C, D, F, G**); 0.5 mm (**E, H, I**).

**Figure 5.**
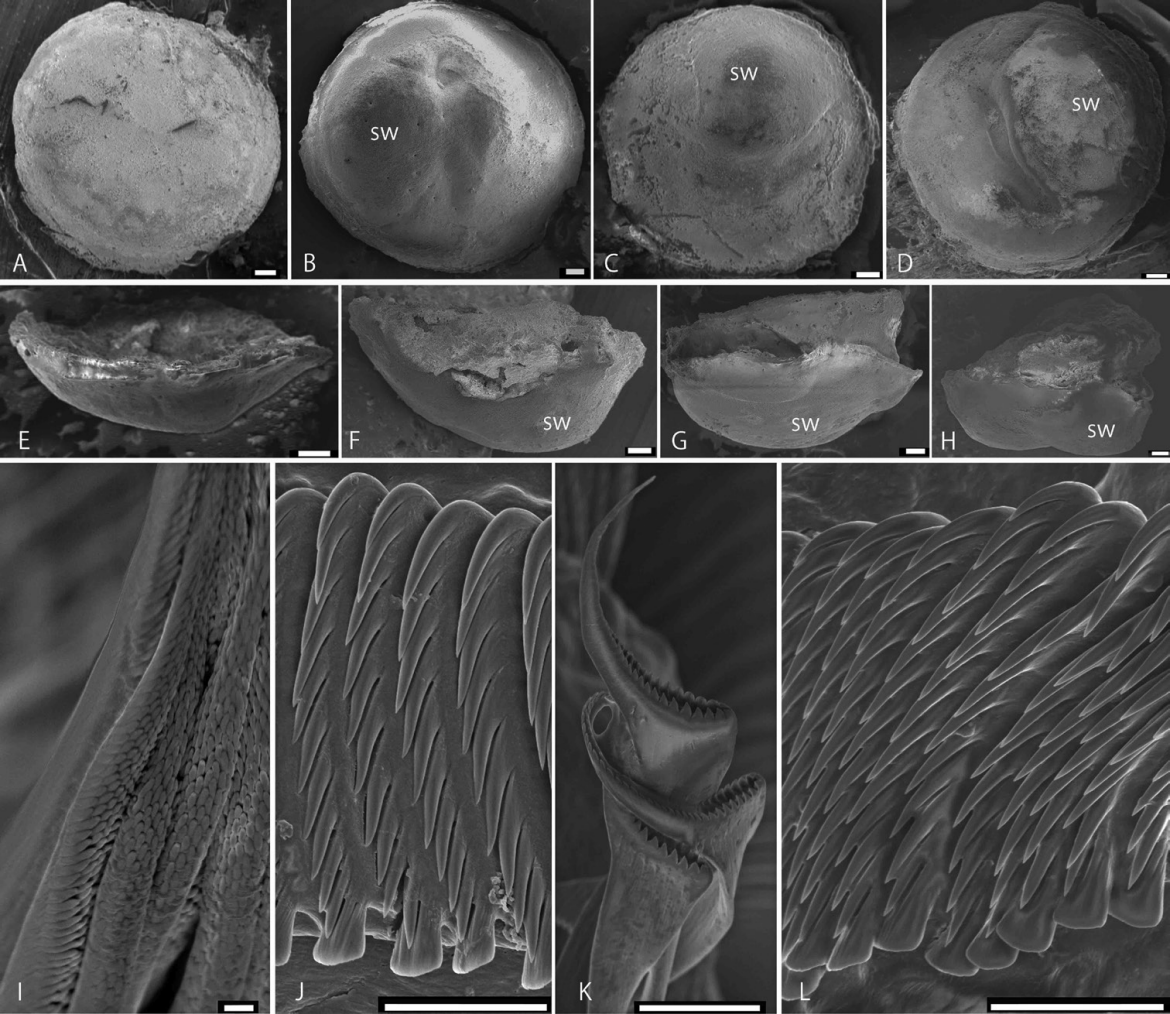
Scanning electron microscopy images of operculum (**A–H**) and chaetae (**I–M**) of *Spirobranchusakitsushima* sp. nov. **A–D** ventral view of endplate **E–H** lateral view. Note that some endplates are with a rounded swelling (sw), **B–D**. **I** thoracic capillary chaetae, scales are in close-up **J** thoracic uncini **K** abdominal true trumpet-shaped chaetae **L** abdominal uncini. Scale bars: 0.1 mm (**A–H**); 0.01 mm (**I–L**).

[Japanese name: Yakko-kanzashi gokai] Figs 2 , 3 , 4 , 5

Pomatoleios crosslandi non Pixell, 1913. — Okuda 1937: 64–67, pl. 2, fig. 1; Onagawa Bay; Utinomi 1956: 41, pl. 21, fig. 3; south of Tohoku.
Pomatoleios kraussii non Baird, 1864. — Imajima and Hartman 1964: 372; Okuda and Imajima 1965: 531; Sawada 1984: 105 [development]; Sawada 1988: 76–77, fig. 5–4, 5–5, 5–6, table 5-3 [reproduction, development]; Imajima 1977: 100–101; Ogasawara Island; 1978: 56; Nii-jima, Izu Islands; 1979a: 178; Kii Peninsula; 1979b: 33; 1984: 165; Oga Peninsula; 1986: 154; Oki Islands; Uchida 1978: 32; Wakayama, Izu, Kochi; Akiyama 1981: 100–101 [distribution, tube characters]; Miura and Kajihara 1984: 343–352; Misaki [distribution, larval development]; Uchida 1992: 369, pl. 71–7; south of central Honshu; Khandeparker et al. 2005; Seto, Wakayama [development]; Horikoshi and Okamoto 2007; Tokyo Bay; Uchida 2008: 180, table 1; Wakayama [distribution].
Pomatoleios kraussii (Baird, 1865)? [sic]. — Imajima 1996: 342, fig. 280; south of Honshu.
Pomatoleios cf. kraussii . — Suzuki et al. 2013: 196, fig. 326.
Spirobranchus kraussii . — Nishi et al. 2017: 96.
Spirobranchus sp. 1. — Kobayashi and Goto 2021: 4–5, figs 2, 3; Wakayama [tube structures, coloration of peduncle, molecular analysis]; Ohno et al. 2021; Echizen, Fukui [distribution].

##### Material examined.

***Holotype***: Japan • Sagami Bay, Kamakura, Wakaejima Island; 35.300628°N, 139.550868°E; 4 June 2020; Nishi, E. leg.; intertidal rocky shore (Figs 1D, 2A, B), collected by hand ; GenBank: LC661622, LC661636, LC661650, LC661664; CBM-ZW 1127, hologenophore.

***Paratypes***: Japan • 4 specimens; same collection data as for holotype; GenBank: LC661623–LC661626, LC661637–LC661640, LC661651–LC661654, LC661665–LC661668; CBM-ZW 1128 to 1131, all hologenophores • 6 specimens, including 1 spec. lacking crown; collection site same as for holotype; 3 August, 2020; GenBank: LC661630–LC661635, LC661644–LC661649, LC661658–LC661663; CBM-ZW 1135 to 1140, all hologenophores • 3 specimens; Shizuoka, Omaezaki (Fig. 1B), Todai-shita; 34.594861°N, 138.225556°E; 4 August 2020; Tanaka, K. leg.; intertidal rocky shore, collected by hand; GenBank: LC661627–LC661629, LC661641–LC661643, LC661655–LC661657, LC661669–LC661671; CBM-ZW 1132 to 1134, all hologenophores.

##### Non-type material.

Japan • 10 specimens; Sagami Bay, Hayama, Chojagasaki; 35.253254°N, 139.578030°E; 8 June 2020; Nishi, E. leg.; intertidal rocky shore, on vertical rocks (see Figs 1E, 2C, D); CMNH-ZW 2273, paragenophores • a single specimen; same collection data as above; CMNH-ZW 2274, paragenophore • a single specimen; same collection data as for paratype from Omaezaki CBM-ZW 1132; CMNH-ZW 2275, paragenophore • a single specimen; same collection data as for paratype from Omaezaki CBM-ZW 1132; MSM-INV-21-1, paragenophore • a single specimen; same collection data as for holotype; CMNH-ZW2276, paragenophore • a single specimen; same collection data as for holotype; CMNH-ZW2277, paragenophore • a colony of worms with tubes; same collection data as for paratype CBM-ZW 1135–1140; MSM-INV-21-2, paragenophore • 3 specimens with tubes; Ogasawara, Chichijima Island, Sakaiura; 27.082548°N, 142.207746°E; 28 June 1995; Nishi, E. leg.; intertidal rocky shore, by hand; CMNH-ZW 2278, syngenophores.

##### Description.

***Tubes*** white, blue, or purple, inside and outside (Fig. 2D, E, G, H, J, K–Q). Tube (sub)triangular in cross-section, with flattened or pointed median sharp keel (Fig. 2E, H, K–N, P), laterally with a row of transverse ridges (Fig. 2K, L, P, Q) and a row of pits below sharp keel (Fig. 2Q). Internal diameter (minimum, mean, maximum) in adults (Fig. 2Q, R, T, U) 1.0, 1.45, 2.1 mm (SD 1.34, *n* = 10 for Kamakura specimens). Outer tube diameter 2.2 to 3.0 mm. A blunt flap over tube mouth (Fig. 2E, M) for 1.5 to 2.8 mm, 1.2 to 2.5 mm wide, in Kamakura and Hayama specimens (see Fig. 2B, D). A sharp flap over tube mouth in Sagami Bay and Yokohama specimens (Fig. 2G, H L, P). Juvenile tube with an undeveloped keel (Fig. 2K). Posterior tabulae rarely found (Fig. 2O).

***Operculum*** with inversely conical to shallow ampulla, covered with calcareous endplate (Fig. 4A–D, F, G) 1.4 mm in diameter (holotype), 1.0–1.5 mm in paratypes, without spines or ornamentations, and usually covered with filamentous algae and bryozoans (Fig. 4C, D, G). Dissected endplate circular in top view, lower part covered with blue membrane (Fig. 4H), bowl-like in lateral view (Fig. 4I). Talon absent (e.g., Fig. 5A, E), slight rounded swellings without bulges or protuberances on underside of calcareous endplates present in some worms (Figs 4H, I, 5B, C, D, F–H). In dissected endplate rounded swelling length 0.38–0.55 mm (Fig. 5B–D, F–H).

***Peduncle*** broad, triangular in cross-section, with simple (unbranched) distal lateral wings (Fig. 4D, F, G) and middle lateral constrictions (Fig. 4F, G, arrowed), rarely branched (Fig. 4E); ventrally with two lateral dark bands on white background (Fig. 4A); lateral wings with alternating pale and dark bands (Fig. 4D, F); inserted at base of radiolar crown left of median line (Fig. 4D, F, G).

***Radioles*** arranged in two semicircles (Fig. 4C, G, J). In type specimens, 17 pairs of radioles in holotype, 13–19 pairs in paratypes. In holotype, radioles 1.6–1.8 mm long, distal tip (without pinnules) 0.3 mm, interradiolar membrane extending 1/2 of radioles (Fig. 4G, H, I). Radiolar eyes 3 or 4 pairs above interradiolar membrane (Fig. 4H, I, K). Mouth palps present.

***Collar and thoracic membranes*.** Collar trilobed, with extensive ventral lobe covering almost entire crown (Fig. 4A–C), wide gap between right and left dorso-lateral lobes (Fig. 4D). Tonguelets folded, leaf-like. Thoracic membranes forming ventral apron across anterior abdominal segment (Fig. 4A–C).

***Thorax*** with six thoracic uncinigerous segments, juveniles with collar chaetae and adults without. Length 2.0 mm in holotype, 1.6–2.5 in paratypes, width 1.0 mm in holotype, 0.7–1.2 in paratypes. Collar chaetae in juveniles simple limbate and with numerous hairlike processes at the base of distal limbate part (*Spirobranchus* chaetae). *Apomatus* chaetae absent. Thoracic chaetae limbate (Fig. 5I). Uncini saw-shaped with 9–11 teeth (Fig. 5J). Ventral ends of thoracic uncinigerous tori widely separated anteriorly, gradually approaching one another toward the end of thorax, thus leaving a triangular depression (Fig. 4A–C).

***Abdomen*** with 46 chaetigers in holotype, 34 to 60 chaetigers in paratypes. Length 3.6 mm in holotype, 3.0–4.0 mm in paratypes. Two or three achaetous segments in anteriormost abdomen (Fig. 4B, C). Uncini saw-shaped with 9–11 teeth (Fig. 5L), incidentally with two teeth above blunt, clearly gouged underneath peg (Fig. 5J). Abdominal chaetae true trumpet-shaped, abruptly bent distally, with two rows of denticles separated by a hollow groove and forming long lateral spine (Fig. 5K). Chaetae becoming increasingly longer posteriorly, but posterior capillary chaetae absent. Posterior glandular pad absent.

***Colour*** oblique lateral stripes of alternating white and gray colors sometimes appearing in opercular peduncles of live specimens (Fig. 4A, D, F, G), these stripes fading in preserved worms. In radiolar crown of worms in Kamakura, Hayama, and Miura Peninsula, the third or fourth of each radiole on dorsal side yellow, particularly above inter-radiolar membrane (Fig. 4A–D, G), whereas some worms lack this yellow coloration. Ventrally, some radioles yellow, but others brown to black, or reddish (Fig. 4F). Radiolar eyes dark brown, pale brown, or dark red (Fig. 4C, D, F, G). Males with creamy white abdomens filled with sperm, females with orange to pale orange abdomens when filled with eggs (Fig. 4B, C).

##### Paleo (sub-fossil) and Recent tube aggregations.

Aggregations of *Spirobranchusakitsushima* sp. nov. were common on vertical natural rocks in Hayama (Fig. 2C, D) from −10 to +15 cm from MSL, while solitary live worms were also found at −100 to +65 cm from MSL. In Kamakura and Tsurugizaki aggregations were abundant on and below natural rocks and in rock pools (Fig. 2A, B, F, G). *Spirobranchusakitsushima* sp. nov. is highly gregarious, sometimes with a density of more than 100 specimens per cm^2^ (Fig. 2C, D, G), and the animals form an intertidal belt on concrete blocks and wall steps, extending horizontally for 10 m along the coast of Yokohama (Fig. 2H–J). At one site in the intertidal of Jogashima, both Recent and sub-fossil tube aggregations were observed within an area of 2 m^2^ (Fig. 3A, D). Recent tubes in densities ranging from 1 to > 100 per 10^2^ were found at −110 to +60 cm from MSL, and dense aggregations (> 10 tubes/10 cm^2^) extending horizontally for ~ 1 m were found at −10 to +20 cm from MSL (Fig. 3D, H). Small patchy paleo-aggregations were found on vertical rock walls and in tide pools (Fig. 3A, B, E–G). The sub-fossil tubes of bluish color were entangled (Fig. 3F, G) and their keels, transversal ridges, and pits, were preserved (Fig. 3E–G).

Both Recent and sub-fossil tube aggregations were also found in Tsurugizaki. The paleo-aggregations (P1 of Fig. 3I, Fig. 1I, north-eastern one) were 25–30 m away in horizontal distance from the Recent aggregations (C1 of Fig. 3I). In P2 and P3 of Fig. 3J, numerous aggregations of fossilized tubes were also found in a marine cave (Fig. 3K, L) 12–15 m away in horizontal distance from the recent aggregation (Figs 1I, 3J). In P2 and P3, sub-fossil tubes were well preserved (Fig. 3K). Particularly in P2, these aggregations were separated into two layers, and upper one found at +150 to +210 cm and the lower one approximately +70 to +100 cm from MSL.

##### Type locality.

Intertidal rocky shore of Kamakura, Sagami Bay, Honshu, Japan.

##### Etymology.

The specific epithet refers to Akitsushima, another name of Japan in the Nara era, ~ 1,300 years ago, as appeared in Kojiki (The Records of Ancient Matters) and Nihon Shoki (The Chronicle of Japan).

##### Taxonomic remarks.

*Spirobranchusakitsushima* sp. nov. is superficially similar to both *S.kraussii* from South Africa and *S.sinuspersicus* Pazoki, Rahimian, Struck, Katouzian & Kupriyanova, 2020 from the Persian Gulf. Pazoki et al. (2020) compared *S.sinuspersicus* and *S.kraussii* in body length, number of abdominal chaetigers, endplate morphology (shape of talon), peduncular wing morphologies and site of peduncular origin, chaetal distribution pattern, and uncinal teeth distributional pattern (rasp- or saw-shaped). The new Japanese species can also be distinguished by endplate morphology, site of origin of peduncle, and uncinal teeth distributional pattern (Table 3). We also compared our new species to two recently described South Asian species, *S.bakau* Sivenanthan, Shantti, Kupriyanova, Quek, Yap & Teo, 2021 and *S.manilensis* Sivenanthan, Shantti, Kupriyanova, Quek, Yap & Teo, 2021 in Table 3; the authority of *S.manilensis* was clarified in Read and Fauchald (2021).

**Table 3.** Comparison of formally described taxa from the *Spirobranchuskraussii* complex. Sizes are in mm.

Characters	*S.kraussii*	*S.sinuspersicus*	*S.lirianeae*	*S.bakau*	*S.manilensis*	*S.akitsushima* sp. nov.
**Total body length**	31 in adults, 9.6–11.7 in juveniles	15 in adults, 2.5–3.5 in juveniles	5 in adults	3–14 in adults	8–18 in adults	5–12 in adults, 2–4 in juveniles
**No. of abdominal chaetigers**	70+10	41+6	~46	27–45	38–41	30–60
**Achaetous abdominal segments**	anterior to middle segments	anterior 1–2 segments	at least first one	anterior 1–3 segments	anterior 1–3 segments	anterior 2–3 segments
**Peduncular lateral wings**	Y-shaped appearance	V-shaped appearance	? V-shaped appearance	? V-shaped appearance	? V-shaped appearance	Y- or V-shaped
**Peduncular wing origin**	Dorso-left of radiolar lobes	Dorso-central of radiolar lobes	Slightly left to mid-dorsal line	left to near medial line	left to near medial line	Dorso-left of radiolar lobes
**Peduncular wing tips**	smooth and pointed	tapering, rarely fringed	rounded	tapering or with truncated	tapering	not fringed, rarely bilobed
**Talon of endplate**	oval, with ~ 10 small protrusions	circular, with 2 or 3 small protrusions	extending into ampulla, basally ending in five rounded teeth	peg-like structure extending into ampulla, terminally bifid or trifid	extending into ampulla, with a series of tooth-like serrations along the edge	absent, no protrusions, or with a rounded swelling
**Thoracic uncini**	saw-shaped	saw- and saw-to-rasp-shaped	saw-shaped	saw-to-rasp-shaped	?	saw-shaped

Imajima (1996: 342, fig. 280) had recorded Japanese “Yakko-kanzashi” as *Pomatoleioskraussii* (Baird, 1865)? [sic!] from around Honshu and to the south of it, with a note stating “uncini shape might be different from the one of South Africa, and thus it might be a different species”. This inference was also noted in Imajima (1997: 23–24). Detailed observations using SEM images of *S.kraussii* uncini and chaetae (Simon et al. 2019) and our new species of Japan (this study) have not shown any differences in morphology and number of teeth of uncini in thorax and abdomen. We distinguish the two species (South African and Japanese) based mainly on the results of genetical analysis and other morphological characters.

Kobayashi and Goto (2021) observed a flap-like structure over the tube mouth in their specimens collected from both Seto, Wakayama and Okinawa. This structure was also observed in the Sagami Bay population of the new species (Fig. 2E, M). The ventral surface of the peduncle in Seto specimens has a dark coloration with dense pigmentation (Kobayashi and Goto 2021) as in ones of our new species from Sagami Bay and Omaezaki (Fig. 4D, F, G). In their Okinawan specimens, the coloration of peduncles was whitish and never heavily pigmented, and lacked lateral banding in some worms (Kobayashi and Goto 2021). As the coloration of Okinawan worms was observed for ethanol preserved specimens, further comparisons of fresh specimens are needed.

*Spirobranchuslirianeae* Brandão & dos Santos Brasil, 2020, another species of the *S.kraussii*-complex from Brazilian waters, has a concave opercular endplate and its talon is with protuberances, while abdominal uncini have 13 or 14 teeth. The subtidal solitary species inhabits tubes with a single sharp longitudinal keel. In *S.akitsushima* sp. nov. the tube has either a flattened projection of the tube keel (Fig. 2E, M) or sometimes a single sharp longitudinal keel (Fig. 2H, L, P), both appearing in the same aggregation. The Japanese new species, while highly gregarious and belt-forming (Figs 2B, C, I, 3D, H), sometimes forms small aggregations and even solitary specimens have been observed. A similar range of appearances, solitary to highly gregarious, was noted and analyzed by Smith et al. (2012) for the New Zealand *S.cariniferus* (Gray, 1843). We summarize the new species characters in Table 3.

*Spirobranchusbakau* Sivananthan, Shantti, Kupriyanova, Quek, Yap & Teo, 2021, recently described from mangrove roots of the Singapore intertidal zone, has very characteristic tubes with wing-like keel structures and in some cases with lateral keels (Sivananthan et al. 2021: fig. 2). Adults of the Singaporean species have collar chaetae, which are limbate type only, no *Spirobranchus*-type chaetae, while thoracic uncini are saw-to-rasp-shaped (Sivananthan et al. 2021). Its opercular talon is a peg-like structure extending downwards from endplate into the opercular ampulla, terminally bifid or trifid (Sivananthan et al. 2021). In contrast, our new species has no wing-like keel structures or lateral keels in tubes, collar chaetae are absent in adults, uncini are saw-shaped, and there is no talon on the underside of the opercular endplate.

*Spirobranchusmanilensis* Sivananthan, Shantti, Kupriyanova, Quek, Yap & Teo, 2021 (non Pillai, 1965), originally described from Manila Bay, Philippines, has also characteristic tubes with white to pale brown color, with one to two keels; peduncle with peduncular wings ending in pointed tips; operculum with sub-triangular talon, extending downwards from endplate into tissue of opercular ampulla, with a series of tooth-like serrations along the edge (Sivananthan et al. 2021). In contrast to this South Asian species, our new Japanese species has a tube with blue coloration (Fig. 2H, L–Q), a median keel (Fig. 2 L, M, P, Q), peduncular wings with rounded tips (Fig. 4F, G, E), and no talon on the underside of the opercular endplate (Fig. 5A–H).

*Spirobranchusakitsushima* sp. nov. has peduncles originating from the left side as in *S.kraussii* (Simon et al. 2019), in *S.lirianeae* (see Brandão and dos Santos Brasil 2020), and in *S.bakau* (see Sivananthan et al. 2021); however, that of *S.sinuspersicus* originates medially (Pazoki et al. 2020). Pazoki et al. (2020) noted the differences in peduncular wings between *S.kraussii* and *S.sinuspersicus*, the former having a Y-shaped, the latter a V-shaped appearance. Judging from the figures of Brandão and dos Santos Brasil (2020: fig. 2B, C, F, G), lateral wings of the peduncle in *S.lirianeae* have a V-shaped appearance. *Spirobranchusakitsushima* sp. nov. has both types of peduncles, which suggests that this character may vary depending on the methods of fixation (e.g., fixed within tubes or without) and necessitates further comparative research.

The upper surface of the endplate is flat and unadorned in all species of the *S.kraussii* complex, but the talon on the lower surface of the endplate appears useful for species delimitation in the complex. The endplate of the new Japanese species is characteristic as it has no talon (= lacking bulges or ornamentations), while other valid species from South Africa, Persian Gulf, Singapore, Brazil, and the Philippines have distinct talons (Simon et al. 2019; Brandão and dos Santos Brasil 2020; Pazoki et al. 2020; Sivananthan et al. 2021). Other as yet not formally described populations of the complex either lack a talon (Sun et al. 2012: Hong Kong) or have one (Bailey-Brock 1987: Hawaii; Belal and Ghobashy 2012: Suez bay). Among them, the population from Suez Bay has a long talon, extending into base of peduncle (Belal and Ghobashy 2012). To clarify the taxonomic status of the above populations of *S.kraussii* complex, a detailed morphological study accompanied by DNA sequence data is warranted.

### ﻿Molecular results

**Figure 6.**
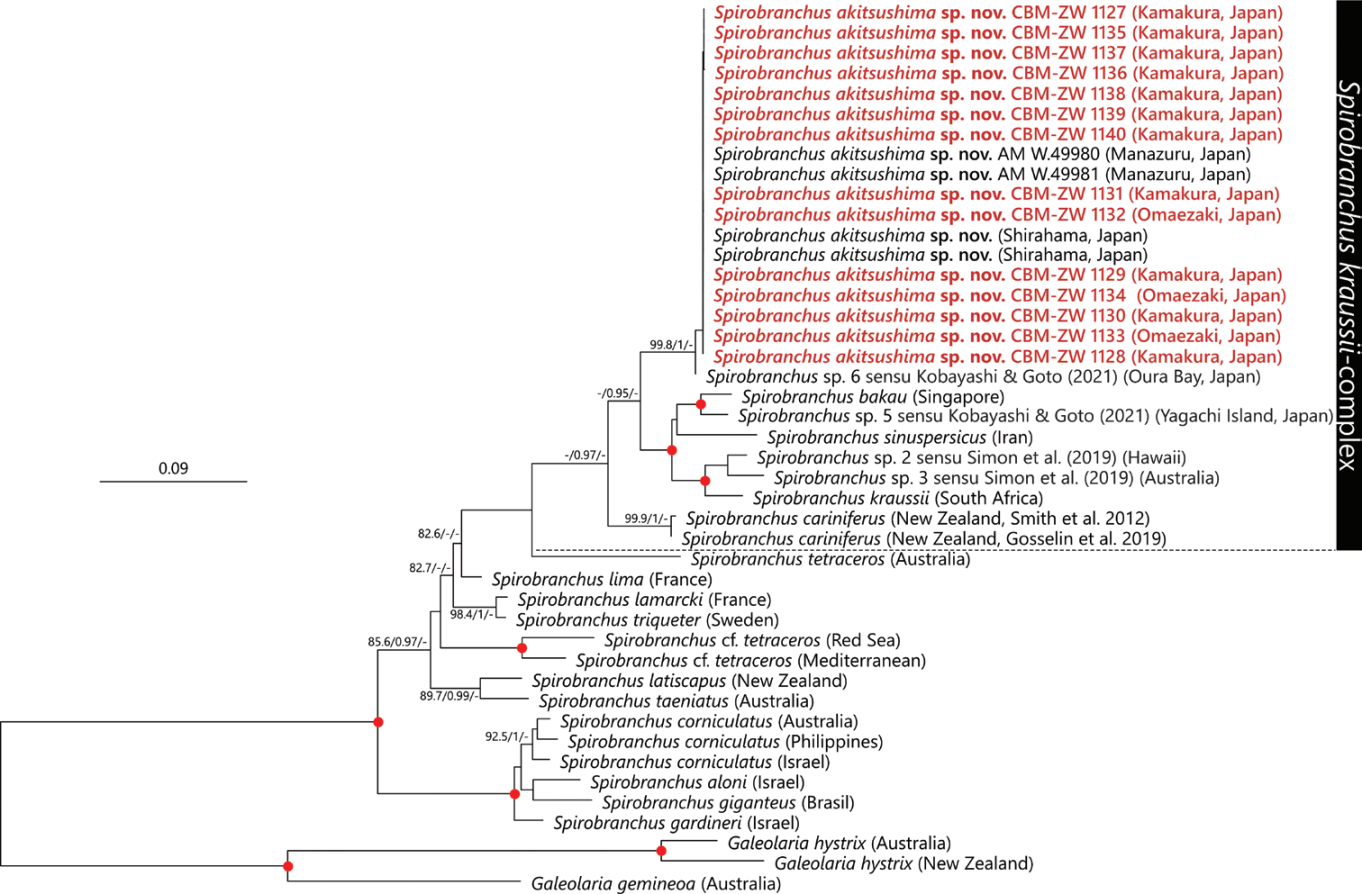
Maximum likelihood tree of *Spirobranchus* species inferred from concatenated gene/region sequence (cytb + ITS2 + 18S + 28S rRNA) obtained from the present study and from DDBJ/EMBL/GenBank (Table 1). The sequences obtained in the present study are highlighted in red. SH-aLRT/approximate Bayes support/ultrafast bootstrap support values of ≥ 80%, ≥ 0.95, ≥ 95%, respectively are given beside the respective nodes. “Red circles at nodes indicate triple high support values of SH-aLRT ≥ 80%, approximate Bayes support ≥ 0.95, and ultrafast bootstrap support ≥ 95%. The scale bar represents the number of substitutions per site. Sequences of *Galeolariahystrix* Mörch, 1863 and *Galeolariagemineoa* Halt, Kupriyanova, Cooper & Rouse, 2009 obtained from DDBJ/EMBL/GenBank were used for outgroup rooting.

In the phylogenetic analysis based on the concatenated dataset (cytb + ITS +18S +28S), the species of *S.kraussii* complex were recovered as a monophyletic clade with high aBayes support (≥ 0.95), but with low SH-aLRT (75.0%) and UFBoot support (62%) values (Fig. 6). *Spirobranchuscariniferus* (Gray, 1843) was recovered as the most basal clade within the complex. *Spirobranchusakitsushima* sp. nov. forms a sister group with *Spirobranchus* sp. 6 sensu Kobayashi and Goto (2021), which is a sister to the clade comprised of *S.kraussii*, *S.sinuspersicus*, *S.bakau*, *S.* spp. 2 and 3 sensu Simon et al. (2019), and *Spirobranchus* sp. 5 sensu Kobayashi and Goto (2021) with high support values (SH-aLRT = 99, aBayes support = 1, UFBoot support = 100).

**Figure 7.**
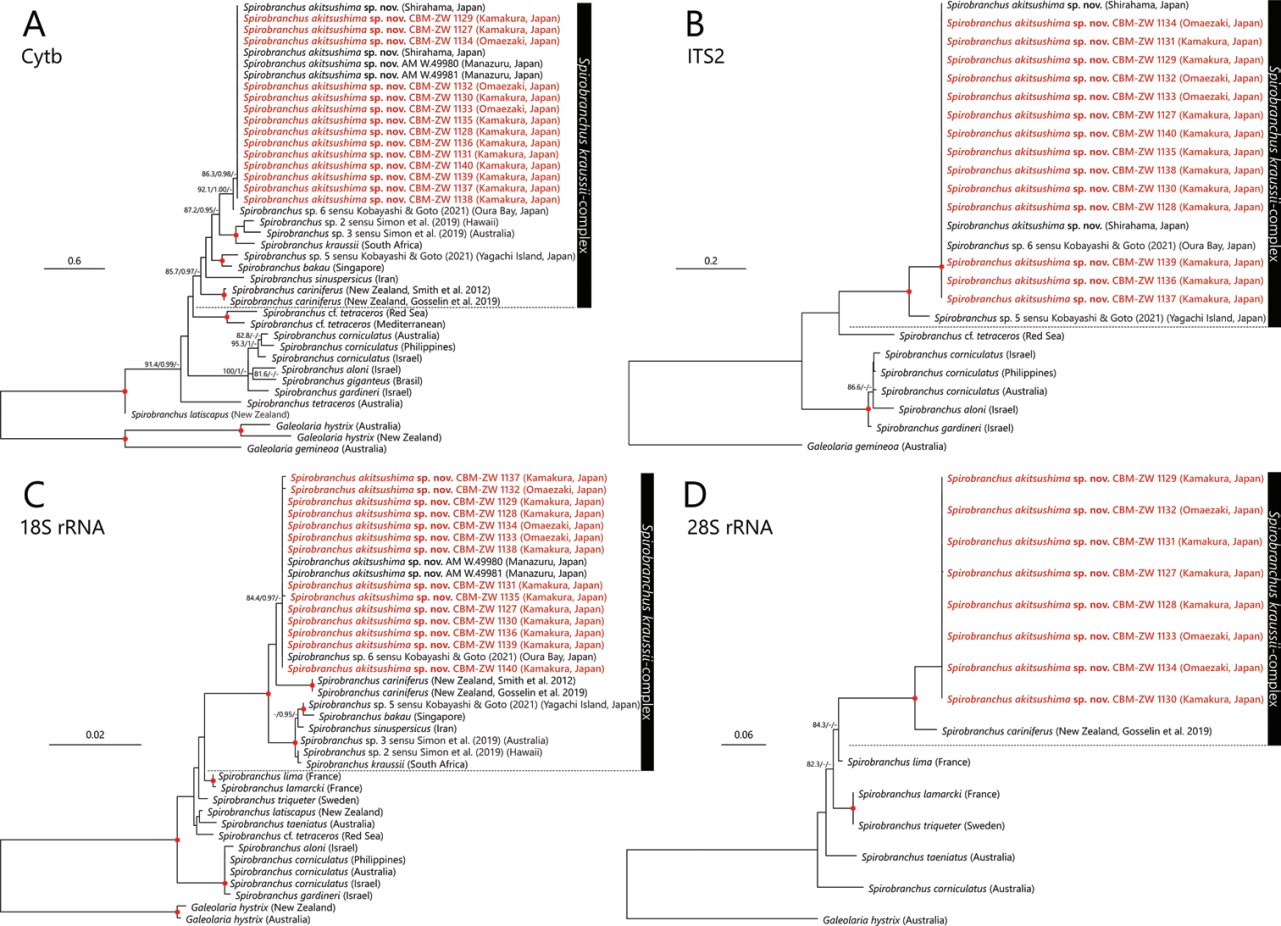
Maximum Likelihood tree of *Spirobranchus* species inferred from mitochondrial cytb (**A**), nuclear ITS2 (**B**), 18S (**C**), and 28S rRNA (**D**) gene/region sequences obtained from the present study and from DDBJ/EMBL/GenBank (Table 1). The gene sequences obtained in the present study are highlighted by red color. SH-aLRT/approximate Bayes support/ultrafast bootstrap support values of ≥ 80%, ≥ 0.95, ≥ 95%, respectively are given beside the respective nodes. Red circles at nodes indicate triple high support values of SH-aLRT ≥ 80%, approximate Bayes support ≥ 0.95, and ultrafast bootstrap support ≥ 95%. The scale bar represents the number of substitutions per site. Sequences of *Galeolariahystrix* Mörch, 1863 and *G.gemineoa* Halt, Kupriyanova, Cooper & Rouse, 2009 from DDBJ/EMBL/GenBank were used for outgroup rooting.

The intra-specific p-distance for cytb sequences of the 18 specimens of our new species was 0.0%. The inter-specific p-distance between the cytb sequences of *S.kraussii*-complex species used for phylogenetic reconstruction in the present study excluding the new species ranged from 14.6–6.9%, with the largest between *S.sinuspersicus* and *S.cariniferus* and the lowest between *Spirobranchus* spp. 2 and 3 sensu Simon et al. (2019) (Table 4). The p-distance between *Spirobranchusakitsushima* sp. nov. and the other *S.kraussii*-complex species ranged from 3.7–24.5%, with the largest p-distance to *S.sinuspersicus* and the lowest to *Spirobranchus* sp. 6 sensu Kobayashi and Goto (2021) (Table 4). *Spirobranchusakitsushima* sp. nov. and *Spirobranchus* sp. 6 sensu Kobayashi and Goto (2021) were 3.7–4.1% different in cytb gene sequence (Fig. 7A, Table 4), but there were no differences in ITS2 region (Fig. 7B) or 18S rRNA gene sequences (Fig. 7C).

**Table 4.** Pairwise distances (p-distance) for cytb sequences between *Spirobranchuskraussii*-complex species used for phylogenetic reconstruction in this study. The p-distances between *S.akitsushima* sp. nov. and the other species are shown as mean values.

	*Spirobranchus* species	1	2	3	4	5	6	7	8	9
1	*S.akitsushima* sp. nov.									
2	*S.* sp. 6 sensu Kobayashi and Goto (2021)	0.038								
3	*S.* sp. 5 sensu Kobayashi and Goto (2021)	0.213	0.217							
4	*S.bakau*	0.201	0.188	0.149						
5	*S.kraussii*	0.221	0.207	0.226	0.205					
6	*S.* sp. 2 sensu Simon et al. (2019)	0.205	0.210	0.235	0.208	0.189				
7	*S.* sp. 3 sensu Simon et al. (2019)	0.198	0.207	0.248	0.234	0.211	0.146			
8	*S.cariniferus* (in Smith et al. 2012)	0.224	0.223	0.218	0.227	0.245	0.261	0.267		
9	*S.cariniferus* (in Gosselin et al. 2019)	0.226	0.228	0.225	0.242	0.252	0.258	0.255	0.018	
10	*S.sinuspersicus*	0.237	0.248	0.248	0.260	0.254	0.251	0.257	0.263	0.269

## ﻿Discussion

In addition to *Spirobranchuskraussii* and *S.cariniferus*, five new species, one from Arabian (Persian) Gulf, one from Brazil, two from South Asia, and the last one from Japan, identifiable mainly by the opercular characters, were recently formally described and named in the *Spirobranchuskraussii* complex (e.g., Brandão and dos Santos Brasil 2020; Pazoki et al. 2020; Sivananthan et al. 2021; this study). *Spirobranchuslirianeae* from Brazil was described without molecular data, and is identifiable by its opercular morphology as well as by its non-gregarious populations inhabiting subtidal habitats. *Spirobranchusmanilensis* from oyster beds of South-East Asia was also described without molecular data, but it is identifiable by opercular morphology (see Table 3).

Live aggregations of *Spirobranchusakitsushima* sp. nov. are common on the shorelines of Sagami Bay and Miura Peninsula, while sub-fossil tube aggregations have also been recorded in Jogashima and Tsurugizaki along Miura Peninsula. The blue- or purple-colored subfossil tubes with prominent characteristic keels and lateral transversal ridges were well preserved (Fig. 3E, G) and stranded ashore well above MSL. The lower one of Tsurugizaki might be a result of the Taisho-Kanto great earthquake in 1923, and the upper one possibly resulted from the Genroku-Kanto earthquake in 1703, as suggested by Nishibata et al. (1988) and Shishikura (2003a, b). It means that we have records of *S.akitsushima* sp. nov. dating from at least 300 years.

Fouling serpulids forming aggregations on artificial substrates are commonly reported as introduced or cryptogenic species (possible introductions) (see Ruiz et al. 2000). Vectors of serpulid introductions are shipping, including hull fouling, and fisheries, including fouling on commercial mollusks such as oysters, scallops, turban shells, and abalones (Ruiz et al. 2000). Highly successful invasive serpulids, such as *Hydroideselegans* (Haswell, 1883) and *H.ezoensis* Okuda, 1934, have been found in large aggregations on ship hulls, a prominent vector of species translocation, and in communities on experimental fouling panels suspended in harbors. These *Hydroides* species have also been frequently recorded on oysters, scallops, and other molluscan shells, another vector of introduction. In contrast, *Spirobranchusakitsushima* sp. nov., although very common on natural substrates, was only reported from unspecified artificial substrates in coastal areas (e.g., Horikoshi and Okamoto 2007) and on concrete blocks of wave breakers and harbor walls in Yokohama harbor (Fig. 2H–J). Specimens of *Spirobranchusakitsushima* sp. nov. have been rarely found on experimental panels (Miura and Kajihara 1983; Raveendran and Harada 2001) and are not found on shells of commercial mollusks. Their distributions are limited to intertidal areas, and Miura and Kajihara (1983) reported that the species appeared 30–80 cm above the mean high water spring tide in Aburatsubo Bay and that the settlement of larvae was not observed on submerged experimental plates. Thus, anthropogenic translocation to other oceans is unlikely to occur. We argue that *Spirobranchusakitsushima* sp. nov. is a species native to Japan, not a non-indigenous species or invader. The species is likely to have regionally restricted distributions around Japan as supported by DNA sequence data and presence of fossilized tube aggregations.

Our molecular phylogenetic analysis using four molecular markers (cytb, ITS2, 18S, and 28S rDNA) has led us to distinguish species among morphologically very similar taxa of *S.kraussii* complex in Japan. The present study showed that the specimens from Manazuru as mentioned by Simon et al. (2019) and other newly sequenced specimens from eastern Sagami Bay and Omaezaki, western-most part of Suruga Bay, belong to the same species described here as *S.akitsushima* sp. nov. This new species is distributed along the Pacific coastline of Honshu from Sagami Bay in the north to Shirahama in the south. The results of molecular analysis suggest that the *S.akitsushima* sp. nov. is genetically distinct from the other *S.kraussii*-complex species described from outside Japan. Interspecific p-distance between the cytb sequences of *S.akitsushima* sp. nov. and the other described *S.kraussii*-complex species were found to be 19.4 to 24.5% (Table 3), which is comparable to that observed within the available members of *S.kraussii*-complex species (14.6–26.9%) and other serpulid genera such as *Ficopomatus* (19.2%, Styan et al. 2017), *Galeolaria* (22.8–24.5%, Halt et al. 2009), and *Hydroides* (15.8–23.1%, Sun et al. 2016).

Kobayashi and Goto (2021) reported three unnamed genetic lineages of *S.kraussii* complex in Japan: *S.* sp. 1 (= *S.akitsushima* sp. nov.) from Seto, Wakayama, southern Honshu and two from Okinawa, *S.* sp. 5 from Yagachi and *S.* sp. 6 from Oura Bay. The presence of two distinct species of the complex in Japan was expected because of the boundary between Osumi Islands and Ryukyu Islands, known as Tokara Tectonic Straight or Tokara Gap, where the Kuroshio current crosses the Ryukyu Islands chain from the west to the east (see Motokawa 2017). As expected, *Spirobranchus* sp. 5 showed a 20.7–22.2% differences in cytb gene sequences with *S.akitsushima* sp. nov. and *S.* sp. 6. Such distance is commonly found between morphologically distinct congeneric species (e.g., Willette et al. 2015; Pazoki et al. 2020) leading Kobayashi and Goto (2021) to the conclusion that *Spirobranchus* sp. 5 is a genetically and ecologically distinct undescribed species.

The status of *Spirobranchus* sp. 6 sensu Kobayashi and Goto (2021) is less certain. Unexpectedly, it is genetically closer (3.7–4.1% only in cytb) to *S.akitsushima* sp. nov. from Honshu than to *S.* sp. 5 also from Okinawa (21.7% in cytb). Kobayashi and Goto (2021) suggested that the genetic differences between Honshu and Oura Bay are quite large, considering the lack of genetic differentiation for specimens within Honshu Island or low genetic diversity at each studied locality. They also noted that “either interbreeding still exist between the lineages in Shirahama and Oura Bay, or that the sorting of the two lineages is incomplete” (Kobayashi and Goto 2021: 13). Clearly, we need to study the population structures of Amami Archipelago and Kyushu situated between Honshu and Okinawa Islands before we can determine whether or not specimens of *S.akitsushima* sp. nov. and *S.* sp. 6 belong to the same species.

Future genetic studies of these Japanese and other Asian populations (e.g., Paik 1989: Korea; Sun and Yang 2014; Huang et al. 1992: China; Sun et al. 2012: Hong Kong) might reveal other distinct species from *S.kraussii* complex.

## Supplementary Material

XML Treatment for Spirobranchus

XML Treatment for Spirobranchus akitsushima
